# Compliance with standard precaution of infection prevention practice and associated factors among health care workers in Ethiopia: Mixed method study

**DOI:** 10.1002/hsr2.830

**Published:** 2022-09-13

**Authors:** Alebachew Kassa, Sisay Eshete Tadesse, Fasil Walelign, Natnael Kebede

**Affiliations:** ^1^ Haike Health Center Haike Ethiopia; ^2^ Department of Nutrition, School of Public Health, College of Medicine Health Sciences Wollo University Dessie Ethiopia; ^3^ Department of Health Service Management, School of Public Health Wollo University Dessie Ethiopia; ^4^ Department of Health Promotion, School of Public health, College of Medicine Health Sciences Wollo University Dessie Ethiopia

**Keywords:** compliance, factors, health care workers, infection prevention practice, standard precaution

## Abstract

**Backgrounds and Aims:**

In developing countries, most infections can be prevented with relatively inexpensive infection prevention methods. However, there is limited information on standard precautions for infection prevention practices among health workers in Ethiopia Therefore, this study aimed to assess the compliance with the standard precaution of infection prevention practice and associated factors among health care workers (HCWs) using a mixed method study.

**Methods:**

A hospital‐based mixed‐methods study design (concurrent mixed method design) was conducted among 378 randomly selected health professionals. Self‐administered questionnaire; an in‐depth interview and an observational checklist were used to collect the data. The collected data were cleaned and entered into Epi data and analyzed using a static package for social science. Descriptive statistics were conducted and the result was reported using frequency, and percentile. Logistic regression was performed to identify associated factors. Adjusted odds ratios with 95% confidence intervals (CIs) and *p* < 0.05 were used to explain statistically significant associations.

**Results:**

The proportion of standard precaution practice among HCWs at Dessie specialized and comprehensive hospital was 55.6% (put the 95% CI). Age ≤ 25 years (AOR = 0.13, 95% CI: [0.04, 0.42]) and age 31 years above age ≤ 31 years (AOR = 0.06, 95% CI: [0.02, 0.3]), positive attitude toward the standard precaution (AOR: 6.43, 95% CI: [3.47, 11.94]). Access to IP guidelines (AOR: 3.13, 95% CI: [1.61, 6.07]). Training on standard precautions (AOR: 3.61, 95% CI: [1.75, 7.48]) were factors associated with standard precaution practice.

**Conclusions:**

In this study, the overall proportion of HCWs' compliance with standard preventive practice was low. HCWs aged 31 years and above, training on standard precaution practice, availability of guidelines in each ward, attitude toward standard precaution practice, knowledge about standard precaution practice, and accessibility of standard precaution supplies were associated with compliance to standard precaution practice. Therefore, the strategies should be designed to fulfill hospitals with supplies, training, and avail guidelines in each ward.

## 1 INTRODUCTION

Standard precautions are a set of infection prevention measures designed to prevent diseases that can be spread through contact with blood, body fluids, broken skin (including rashes), and mucous membranes.^1^ Standard precautions are the work practices needed to achieve the highest level of infection control for the treatment of all patients, regardless of diagnosis. It refers to all policies, procedures, and activities designed to prevent or minimize the risk of the spread of infectious diseases in healthcare settings.^2^, ^3^ Adherence to basic infection prevention and control practices is critical, not only in acute care hospitals but in any setting with limited infection prevention infrastructure.^4^ Health care professionals come into contact with blood and other bodily fluids during their work.

Globally, approximately 3 million health care professionals have percutaneous exposure to bloodborne pathogens; 2 million for hepatitis B virus (HBV) and 900,000 for hepatitis C virus (HCV), and 170,000 for human immune deficiency virus (HIV) each year. more than 90% of infections occur in developing countries.^5^ Hospital‐acquired infections worldwide are a major public health problem, leading to increased morbidity, mortality, and health care costs. Hospitals are a major source of infection risk when providing healthcare services.^6^

The prevalence of healthcare‐associated infection in teaching hospitals in Ethiopia was 14.9%.^4^ Healthcare‐related infections affect patients, visitors, family members, and health care workers (HCWs). Patients are more susceptible to hospital‐acquired infections due to invasive procedures.^7^ Compliance with infection prevention and control practices is important to provide safe and high‐quality patient care across all settings where healthcare is delivered.^8^

Despite the implementation of different intervention strategies, such as hand hygiene, personal protective equipment (PPE), disinfection and sterilization, injection safety, and proper waste disposal, adherence to standard precautions among health workers is low.^9^, ^10^ the main reasons for low compliance are the unavailability and inaccessibility of PPE; insufficient knowledge and attitudes toward standard care (SP); less administrative support for safe labor practices; feedback on HCW safety performance, workplace safety, work location, job category, and marital status.^11^, ^12^ Previous studies were conducted using a cross‐sectional study design with limited qualitative methods and were unable to see the experience of health care professionals adhering to the standard of care. conducting with a mixed method is more important to get multiple determinants and comprehensive results for intervention.^2^, ^4^ Therefore, this study aimed to assess the compliance with the standard precaution of infection prevention practice and associated factors among HCWs using a mixed study method. The result of this study will be used for program planning to improve compliance with standard precaution infection prevention practices among HCWs.

## 2 MATERIALS AND METHODS

### 2.1 Study design and period

A hospital‐based mixed‐methods study design (concurrent mixed method design) was conducted from March to April 2021. concurrent mixed method design concurrently collected the data for both quantitative and qualitative method studies.

### 2.2 Population

All HCWs who have been working at Dessie comprehensive and specialized hospital were taken as the source population. All HCWs who were working at Dessie comprehensive and specialized hospital during the data collection period were considered as the study population.

### 2.3 Inclusion and exclusion criteria

All HCWs who were involved in clinical services during the study period had direct contact with patient care including, residency medical training cleaners and housekeepers were included in the study. While those who have been working in the administration offices were excluded from the study.

### 2.4 Sampling method and sample size determination

The sample size was determined by using a single population proportion formula by taking the following assumptions: prevalence of standard precaution from a study conducted in Hawassa comprehensive and specialized Hospital 56.5%,^9^ a confidence interval (CI) of 95%, marginal error of 5%.^9^

$$n=\left({\left(1.96\right)}^{2}\times 0.565\left(1-0.565\right)\right)/{\left(0.05\right)}^{2}=378.$$

Finally, by adding 10% nonresponse, the total sample size was 415.

The sample size for the qualitative method was determined by the degree of saturation.

**Figure 1:**
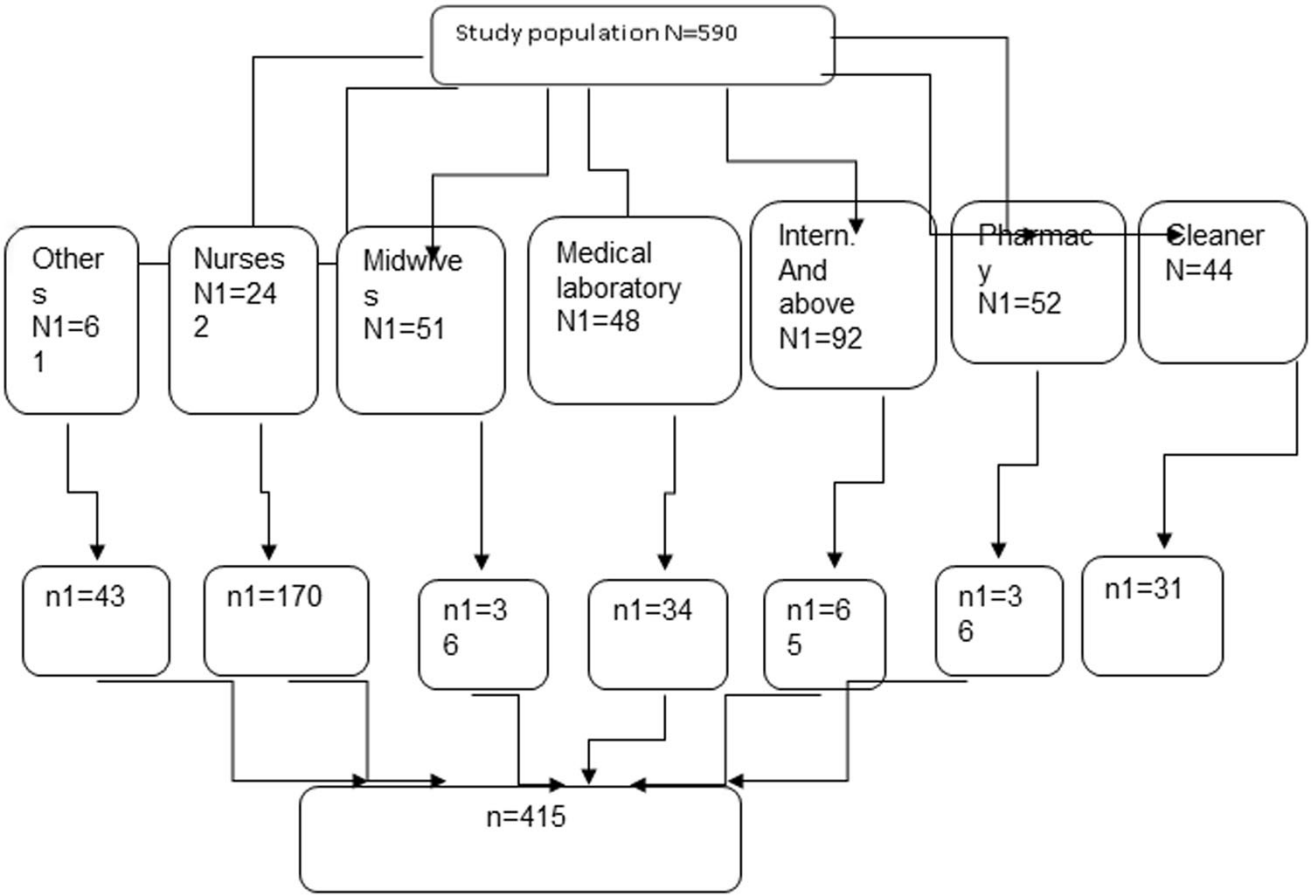
Sampling procedure to compliance with standard precaution of infection prevention practice among health care workers at Dessie comprehensive and specialized hospital, 2021

A stratified random sampling method was used to select study participants First the sample size was proportional allocated for each health profession (cleaner, pharmacy, intern students and above, medical laboratory, midwives, nurses, and others) based on their number and followed by simple random sampling from each stratum. Purposive sampling was used for the qualitative study (Figure 1).

### 2.5 Data collection procedure and quality assurance

The questionnaire was initially developed in English by reviewing available literature and Ethiopian infection prevention and control (IPC) guidelines. The training was given to data collectors and a supervisor. A pretest was done at Boru general hospital.^9^ The data collection tool included 24 compliance with standard infection prevention, which was measured by three points Likert scale questionnaires (1 = never, 2 = sometimes, 3 = always)^1^; seven sociodemographic questions; seven institutional‐related questions; 13 knowledge‐related questions, and 14 attitudes toward standard precaution related questions.

Qualitative data was collected by using an in‐depth interview and observational checklist. Interview guide questions had simple definition terms of the broader contextual definitions to interlink the ideas (Supporting Information).

### 2.6 Data processing and analysis

Data were checked, entered, and cleaned using Epi data version 4.6.1 and analyzed by SPSS version 25 for further analysis. Descriptive analysis was done and the result was reported using proportions, percentages, frequency distribution, and mean + standard deviation.

Binary logistic regression was used to examine the relationship between the proposed predictors and compliance with the standard precaution of infection prevention. Variables with a value less than 0.2 in the bi‐variable analysis were entered into a multivariable binary logistic regression to identify the independent factors associated with compliance with the standard precaution of infection prevention. Adjusted odds ratio with a 95% CI and a *p* < 0.05 were used to declare the statistically significant association. The model fitness was checked by Hosmer and Lemeshow.

Qualitative data were analyzed using thematic content analysis. Before analysis, all the collected data were transcribed into English. Transcription of data was done, then manually narrated, summarized, and analyzed thematically. Coding was conducted carefully and read line by line several times by the principal investigator. The codes were grouped into categories and then analyzed based on thematic content analysis. Finally, the quantitative finding was supplemented by qualitative findings with triangulation.

## 3 RESULTS

### 3.1 Sociodemographic characteristics of participants

Three hundred and ninety‐six HCWs participated in this study. The mean age of the study participant was 29.82 ± 4.0 years. More than half of the respondents (54.3%) were females. slightly lower than three‐fourths (71.70%) were married. One in five (42.90%) of the respondents were nurses. More than half (54.53%) of the respondents had a work experience of 5 years and above. Regarding their educational status, 227 (57.34%) of the respondents were first‐degree holders (Table 1).

**Table 1:** Sociodemographic factors of healthcare workers in Dessie comprehensive and specialized hospital, North East Ethiopia, 2021

Variables		Frequency and percent of the respondent	
		Frequency	%
Age*	≤25 years	55	13.89
	26–30 years	186	46.97
	≥31 years	155	39.14
Sex	Male	181	45.71
	Female	215	54.29
Marital status	Single	99	25.00
	Married	284	71.72
	Divorced	13	3.34
Profession	Medical doctor	50	12.60
	Medical laboratory	34	8.62
	Nursing	170	42.93
	Midwifery	36	9.12
	Pharmacy	36	9.14
	Cleaner	30	7.65
	Other	40	10.16
Educational status	Diploma	103	26.00
	First degree	227	57.30
	Second degree	39	9.81
	Other	27	6.82
Experience*	≤2 years	49	12.43
	3–4 years	131	33.03
	≥5 years	216	54.54

*Note*: Socio demographic characterstics is important as modifying factors and background variable for others variables.

### 3.2 Level of compliance with the standard precaution of infection prevention practice

This study showed that 220 (55.6%) of the respondents had good compliance with the standard precaution of infection prevention practice. Of those 64.6% had the highest compliant do not bent needles and the least compliant were 35.4% of wear eye goggles when indicated also of observed participants, 52.10% had good compliance with IP practice.

### 3.3 Health institution‐related factors

Among 396 study participants, 222 (56.1%) had infection prevention supplies within the institution. More than half 223 (56.3%) of them were having standard precautions guidelines and 236 (59.3%) were having monitoring and evaluation on standard precautions (Table 2).

**Table 2:** Institutional factors of the study participants in Dessie comprehensive and specialized hospital, North East Ethiopia, 2021

Institutional factors		Frequency	Percent (%)
Availability of supplies	No	174	43.9
	Yes	222	56.1
Availability of guidelines	No	160	40.4
	Yes	236	59.3
Monitoring and evaluation	No	173	43.7
	Yes	223	56.3

### 3.4 Individual related factors

Two hundred and thirty‐seven (59.3%) of the respondents knew the standard precautions of IPC practice, 172 (43.4%) were having training about standard precautions of IPC practices, and 193 (48.7%) were having a positive attitude toward standard precautions of IPC practices (Table 3).

**Table 3:** Individual factors of the study participants at Dessie comprehensive and specialized hospital, North East Ethiopia, 2021

Individual factors		Frequency	Percent (%)
Knowledge about SPs	No	159	40.2
	Yes	237	59.8
Training about SPs	No	224	56.6
	Yes	172	43.4
Attitude toward SPs	No	203	51.3
	Yes	193	48.7

### 3.5 Hand hygiene compliance

According to this study, among the HCWs 182 (46%) always wash hands before touching a patient and 200 (50.5%) of HCWs always wash hands before clean/aseptic procedures. Among the respondents, 243 (61.4%) wash their hands after touching body fluid exposures, and 191 (48.2%) of HCWs always wash their hands after touching a patient. lower than half 177 (44.7%) of the study participants wash their hands immediately after removing gloves, 168 (42.4%) wash their hands between patient contact and 172 (43.4%) always wash their hands touching patient surroundings.

Of the 140 HCWs who received standard preventive IP practice, 97 (69.3%) washed their hands with soap and water or used alcohol‐based hand sanitizer before and after surgery, and 95 (67.9%) Before hand washing and removing gloves, 131 (93.6%) observed that participants washed their hands after handling contaminated items, and 89 (63.6%) performed hand hygiene before preparing the medication.

#### 3.5.1 Use of PPE

Of the respondents that self‐reported mostly 236 (59.6%) avoided wearing the gown out of the workplace and the least 166 (41.9%) compliant had about wear eye goggles when indicated. The finding of compliance with PPE by medical staff at Dessie Hospital and specialist hospitals is described (Table 4). Of the observed HCWs, nearly all 133 (95%) wore protective clothing during procedures with potential exposure to blood or bodily fluids and procedures with the potential for splashes of blood or other bodily fluids, but fewer than 94 (67.1%) used mouth, nose, and eye protection.

**Table 4:** Level of compliance with personal protective equipment among health care workers at Dessie comprehensive and specialized hospital, 2021

Compliance variable	Respondent		
	Never	Sometimes	Always
Protect myself against body fluids of all patients regardless of their diagnosis	13 (3.3%)	163 (41.2%)	220 (55.6%)
Provide care considering all patients as potential infectious	24 (6.1%)	154 (38.9%)	218 (55.1%)
Wear clean gloves whenever there is possibility of any body fluids	4 (0.8%)	156 (39.6%)	236 (59.6%)
Avoid wearing the gown out of work palace	9 (2.3%)	148 (37.3%)	239 (60.4%)
Wear a waterproof apron whenever there is a possibility of body fluid	36 (9.1%)	194 (49.0%	166 (41.9)
Wear eye goggles when indicated	37 (9.3%)	219 (55.3)	140 (35.4%)
Wear mask when indicated	12 (3.1%)	178 (44.9%)	206 (52%)
Wear boots when indicated	39 (9.8%)	205 (51.8%)	152 (38.4%)

### 3.6 Level of compliance with health care waste management and sharp safety of standard precaution

Of 396 study participants, 256 (64.6%) did not bend the needles and the least 176 (44.4%) did not recap the needles.

### 3.7 Instrument processing and waste management

Of the 140 health workers observed, 109 (77.9%) used instruments that were decontaminated immediately after use by soaking the instruments in 0.5% chlorine for 10 min, then washing them in soapy water, and then rinsing in clean water and drying before sending it to high‐grade for disinfection or sterilization. Three‐quarters (74.3%) of the 104 participants observed did not recap or bend the needle after use, and both needles and syringes were immediately discarded in puncture‐resistant containers. Of the observed participants, 94 (67.1%) had solid waste separated at the point of use. At least 96 (66.4%) stab‐resistant sharps containers are 3/4 full by category.

### 3.8 Factors associated with compliance with the standard precaution of IP practice

The bi‐variable logistic regression analysis result showed that infection prevention guidelines, training, work experience, the attitude of respondents toward standard precaution, standard IPC supplies, knowledge of the respondents toward IP practice, educational level, profession, department (unit), age, and sex were identified as candidates for multivariable logistic regression analysis. In multivariable binary logistic regression analysis, age ≤ 25 years (AOR = 0.13, 95% CI: [0.04, 0.42]) and age 31 years above age ≤ 31 years (AOR = 0.06, 95% CI: [0.02, 0.32]), positive attitude toward standard precaution (AOR: 6.43, 95% CI: [3.47, 11.94]), access to IP guidelines (AOR: 3.13, 95% CI: [1.61, 6.07]). training on standard precautions (AOR: 3.61, 95% CI: [1.75, 7.48]) and participants who knew standard precautions of IPCs were three times more likely to comply with standard precaution as compared to those who did not know (AOR with 95% CI) (Table 5).

**Table 5:** Multivariable analysis result by binary logistic regression for factors affecting compliance with standard precaution of health care workers at Dessie comprehensive and specialized hospital, 2021

Variable		Compliance with standard precaution of IPCs		Crude odds ratio with 95% CI	Adjusted odds ratio with 95% CI
		Not compliant (*N*= 176)	Compliant (*N* = 220)		
Sex	Male	73	108	1	‐
	Female	103	112	0.74 (0.49–1.10)	1.28 (0.70–2.36)
Age	25 years and below	26	29	1	1
	26–30 years	95	91	0.86 (0.47–1.57)	0.13 (0.04–0.42)*
	31 years and above	55	100	1.63 (0.87–3.04)	0.06 (0.02–0.33)*
Educational status	Diploma	49	54	1	1
	First degree	99	128	1.17 (0.74–1.87)	1.59 (0.78–3.23)
	Second degree	7	32	4.15 (1.68–10.25)	5.34 (1.40–20.37)^a^
	Others	21	6	0.26 (0.08–0.70)	0.00
Profession	Doctor	25	25	1	‐
	Laboratory	15	19	1.37 (0.57–3.30)	2.34 (0.55–10.02)
	Nurse	48	112	2.54 (1.33–4.86)	4.02 (1.31–12.31)^a^
	Midwifery	22	14	0.71 (0.30–1.72)	1.13 (0.21–6.06)
	Pharmacy	20	16	1.37 (0.57–3.30)	2.49 (0.09–73.16)
	Cleaner	24	6	0.26 (0.09–0.72)	0.00
	Other^b^	22	18	0.81 (0.35–1.87)	0.24 (0.02–2.45)
Department	Obs/gyn	41	32	1	‐
	Medical	29	48	1.02 (0.46–2.28)	0.55 (0.17–1.83)
	Surgical	25	38	2.17 (0.98–4.82)	0.41 (0.12–1.41)
	Pharmacy	20	16	2.00 (0.88–4.54)	0.19 (0.01–4.65)
	Laboratory	10	12	1.05 (0.42–2.65)	0.86 (0.19–3.91)
	Emergency	21	20	1.58 (0.55–4.55)	0.20 (0.05–0.76)^a^
	Pediatrics/NICU	9	38	1.25 (0.51–3.05)	1.56 (0.40–6.14)
	Other^c^	21	16	5.54 (2.09–14.69)	2.00 (0.19–21.00)
Experience	≤2 years	35	14	1	1
	3–4 years	59	72	3.05 (1.05–6.20)	2.61 (0.80–8.56)
	5 years and above	82	134	4.09 (2.07–8.05)	5.12 (1.33–19.70)^a^
Availability PPEs	No	104	70	1	1
	Yes	72	150	3.10 (2.05–4.68)	2.23(1.16–4.30)*
Training	No	141	83	1	1
	Yes	35	137	6.65 (4.20–10.53)	3.61 (1.75–7.48)*
Monitoring and evaluation	No	100	60	1	1
	Yes	76	160	3.51 (2.30–5.34)	1.22 (0.62–2.40)
Knowledge	No	111	65	1	1
	Yes	48	172	6.12 (3.96–9.53)	3.06 (1.61–5.83)*
Attitude toward IP practice	No	128	75	1	1
	Yes	48	145	5.15 (3.343–7.952)	6.43 (3.47–11.94)*
Guidelines	No	114	62	1	1
	Yes	59	161	5.017 (3.265–7.711)	3.13 (1.61–6.07)

Abbreviations: CI, confidence interval; IPC, infection prevention and control; PPE, personal protective equipment.

^a^ The categorized variable was significant but considering the general variable wasn't significant.

^b^ Medical radiology technologist, ophthalmic nurse, psychiatric nurse, environmental health.

^c^ ART/TB ward, physiotherapy ward, psychiatric ward, radiological ward, oncology ward.

* Significant at *p*≤ 0.05.

### 3.9 Key informant interviews

A total of nine key informant interviews involving department heads based on their experience and position were conducted. Among these, almost all agreed that compliance with the standard precaution was influenced by factors. Seven themes emerged from the analysis of the interview into institutional (human resources, IPCs supplies, high traffic flow, management system, and guidelines) and HCWs: emergency (time constraint), training, and HCWs commitment.

#### 3.9.1 Inadequate training

This is a barrier that hinders compliance with standard precaution IPs practice almost. All key informant interviewees (KIIs) explained that IPC guideline was dated but mostly HCWs did not take the training. One of the KIIs stated,

“…I am not trained on standard precaution infection prevention guidelines….” (A 7 years experienced surgical nurse). “Similarly another KIIs mentioned,*”…Continuous basic and refresher training on standard precaution will empower us to comply with the standard precaution*” (6 years experienced optometry nurse).

#### 3.9.2 Unavailability of guidelines

Except for three interviewers, the remained key informant interviewee agreed on the shortage of IPC guidelines. *“…the unavailability of up‐to‐date IPC guideline, we didn't have guidelines and hinder to comply the standard precaution practices”* (8 years experienced medical laboratory technologist).

#### 3.9.3 Weak management system

The majority of the interviewees agreed that there was monitoring and feedback by the hygiene and sanitary officer of the hospital but they didn't use it for decision‐making.

“…*As sanitary officers, we have a monitoring and evaluation system and we give strong feedback to each department. But there is no improvement on compliance of standard precaution practice among HCWs due to weak management system and they didn't use the feedback for decision making*” (A 10 years experienced sanitary officer).

#### 3.9.4 Unavailability of IPC supplies

The majority of the key informant interviewee agreed that there was a shortage of IPCs supplies like pipe running water, soap, alcohol, sanitizer, and PPEs. The unavailability of clean water in their wards was reported by most key interviewees. “*…I didn't wash hands based on WHO hand hygiene guidelines recommendation because our wards didn't have access to piped water and unavailability of alcohol for alcohol‐based hand rub”* (A 7 years experienced midwife). *“…even for the current emerging pandemic COVID‐19, we haven't adequate PPEs like examination glove due to inaccessible widely in our country. This made HCWs not to comply with IPC practices”* (A 8 years experienced pharmacy technologist).

#### 3.9.5 Shortage of human resources

Most of the key informant interviews agreed that shortages of HCWs are obstacles to complying with standard precaution infection prevention practices.

*“…even if the hospital is comprehensive and specialized and serves many patients, It has only 44 cleaners and they have not covered all wards based on standards of IPC guidelines”* (An 11 years experienced cleaner).

#### 3.9.6 Emergency and overwork load

The majority of interviewees verbalized that time constraints and high patient flow impose a high burden to practice standard precautions. One participant states that; *“…the reason for not complying with the standard precaution of IPCs practice is a high case flow at an emergency ward makes us not to practice the standard precaution. In this ward, we aim to save lives and reduce patient waiting time”* (A 8 years experienced BSc nurse).

#### 3.9.7 Lack of commitment

Almost all key informant interviewees verbalized that some HCWs were exposed to sharp needle injuries due to a lack of HCWs' commitment to the segregation process. “…*in the last 3 months around 45 HCWs were exposed to needle prick injury due to hospital HCWs segregation problem*” (A 10 years experienced sanitary officer).

## 4 DISCUSSION

The overall finding of compliance with the standard precaution of IP practice among HCWs was 55.6%. This finding was consistent with studies done in Hawassa comprehensive and specialized hospital 56.5%^9^ and Gondar and Felege Hiwot hospitals 55%.^4^ This similarity might be due to the similar sociodemographic (professions, gender age group, etc.) and socioeconomic backgrounds of the study participants. While it was lower than studies conducted in the Dawuro zone was 65% and Addis Ababa was 66.1%.^2^, ^13^ It was high compared with the result from the Wolayta zone 42.4%, Mizan Tapi general hospital t 46.8%, and Mekelle special zone 42.9%.^14^, ^15^ This discrepancy might be due to the type of healthcare facilities, sample size, study setting, and fear of being exposed for the pandemic disease of COVID‐19.

The current result found statically significant between compliance and getting training on standard precautions of IPs. HCWs that had training on standard precautions were nearly four times more likely to comply as compared to those who did not take training on standard precautions. This finding is also supported by the key informant interviews. *“…Continuous basic and refresher training on standard precaution will empower us to comply with the standard precaution*.” The result of this finding is consistent with studies conducted in West Arsi district, Wolayta zone, and Hawassa comprehensive and specialized hospital in which HCWs who get training on standard precaution had good compliance.^16^, ^17^ This could be because training will equip health care providers with good knowledge and skill to practice standard precautions.

The study found that HCWs with an IP policy were three times more likely to follow standard precautions than those without IP guidelines. This finding is also explained by the key informant interview result, “…*unavailability of up‐to‐date IPC guideline will hinder the compliance of HCWs to standard precaution practices*.” This result is consistent with studies conducted in Hawassa and Gondar^9^, ^10^ in which HCWs who had guidelines were having good compliance than those without guidelines. This may be because the presence of guidelines will encourage health care providers to practice standard precautions.

In this study, a positive attitude toward standard precaution was six times more likely to have compliance than those who had a negative attitude toward standard precaution. This result is in line with the findings of studies done in the Hadiya zone and Gondar comprehensive and specialized hospital, which revealed that HCWs who have a good attitude toward standard precaution had good compliance than those who had negative attitudes toward standard precaution.^10^, ^18^ This may be because of the strong commitment and fear of nosocomial infection.

In this study, participants who had access to standard precaution materials were two times more likely to comply with the standard precaution of infection prevention practice than those who didn't have access to IPs materials. Also, this finding is supported by the result of key informant interviews. *“…I didn't wash hands based on WHO hand hygiene guideline recommendation because our wards don't have access to piped water and unavailability of alcohol for alcohol‐based hand rub”* (A 7 years experienced midwife). This finding is in line with the studies conducted in Dawuro zone and Gondar specialized and comprehensive hospitals in which HCWs who had availability of IPs supplies were having good compliance than HCWs who had no IP supplies.^2^, ^10^

HCWs in the age range of 26–30 years were 87% less likely to comply than those with age ≤25 years and those aged 31 years above were nearly 94% less compliant than those with age ≤25 years. This is incongruent with a study done in the Dawuro zone, which revealed that younger age HCWs had poor compliance than older age.^2^ This difference may be because of the study setting, sample size, and recent memory. Social desirability bias is a limitation of the qualitative study since its data collection method was an in‐depth interview that is the interviewer administers guidelines.

## 5 CONCLUSIONS

In this study, HCW's overall compliance with standard precautions was low as compared to other studies. HCWs who had a positive attitude toward standard precaution, knowledge toward standard precaution, training on standard precaution, younger age, availability of guidelines in the ward, and availability of IPs supplies were factors associated with the compliance. And also factors that influence compliance with the standard precaution of IPCs practice were IPCs supplies, human resources, training, commitment, management system, guidelines, and workload. Therefore, to increase the compliance of HCWs with standard precaution, continued training will be given to HCWs on standard precaution practice, preparation and distribution of standard precaution guidelines for all health facilities, avail infection prevention supplies, and regular strengthening and monitoring will be done.

## AUTHOR CONTRIBUTIONS

**Alebachew Kassa**: Conceptualization; Formal analysis; Investigation; Methodology. **Sisay Eshete Tadesse**: Conceptualization; Formal analysis; Methodology; Writing–original draft; Writing–review & editing. **Fasil Walelign**: Conceptualization; Formal analysis; Methodology; Writing–original draft; Writing–review & editing. **Natnael Kebede**: Conceptualization; Formal analysis; Writing–original draft; Writing–review & editing.

## CONFLICT OF INTEREST

The authors declare no conflict of interest.

## ETHICS STATEMENT

Ethical clearance was obtained from the Institutional Review Board of the school of Public health, College of Medicine and Health Sciences at Wollo University. After explaining the purpose of the study, written informed consent was obtained from participants before data collection. They were informed that participating in the study was voluntary and their right to withdraw from the study at any time during the interview was assured. For this purpose, a one‐page consent letter was attached as a cover page of each questionnaire stating the general objective of the study and issues of confidentiality.

## TRANSPARENCY STATEMENT

The lead author Natnael Kebede affirms that this manuscript is an honest, accurate, and transparent account of the study being reported; that no important aspects of the study have been omitted; and that any discrepancies from the study as planned (and, if relevant, registered) have been explained.

## Supporting information

Supporting information.

## Data Availability

All the necessary data are included in the manuscript. An English version data collection tool and detailed operational definitions of the outcome variable are accessible on a reasonable request from the corresponding author.
