# Supplementary material for: Compliance with standard precaution of infection prevention practice and associated factors among health care workers in Ethiopia: Mixed method study
Source: Health Sci Rep. 2022 Sep 13;5(5):e830. doi: 10.1002/hsr2.830 (PMC9470010; doi:10.1002/hsr2.830)
Supplement: Supplementary file 1 — Supporting information. [file HSR2-5-e830-s001.doc]

APPENDIXES

## Annex I: English Consent /assent form and Information sheet

Information sheet

Good morning/afternoon, my name is_________________________ and I am health professional working in_______________ I am also a part of team carrying out this study

Study Title፡ Compliance with standard precaution of infection prevention and associated factors among health care workers in Dessie comprehensive specialized hospital , South Wollo, Ethiopia.

Name of the Organization: School of public health, College of Medicine and Health Sciences, Wollo University.

1) Purpose of the Research Project: The aim of this study is to assess Compliance with standard precaution of infection prevention and associated factors among health care workers in Dessie comprehensive specialized hospital , South Wollo, Ethiopia. In line with this, the findings will also provide baseline information for health sector administrators, concerned bodies and the study participants themselves in complying to standard infection prevention, that further reduce the morbidity and mortality associated with hospital acquired infection .

2) Procedure: As study participants fulfill the criteria, they will kindly be requested to give genuine answers to the prepared questionnaire.

3) Risk and discomforts: they will not face any physical or psychological risk and no damage resulting from the research procedures. Simply you will give answer the question.

4) Benefits of the study: This study will have a great value onhealthcare workers, healthcare facility managers, researchers, policymakers and other stakeholders as appropriate.

5) Compensation for participation: you will not receive any payment for your participation in this research study.

6) Confidentiality of your information: All information gathered from the study participant will remain confidential. Your participation in this study is strictly anonymous. Personal information will be treated confidentially and no any circumstances it will be transmitted to any person or organization.

7) Right to refusal or withdraw: your participation in the study is voluntary; you have full right to refuse from participating in this research. You can refuse to give sample and not to respond any or all the questionnaires and this will not affect them on using any kind of services from the institutions.The questionnare will be taken 25 minutes.

8) Person to Contacts: Ethical clearance review committee of School Public Health, College of Medicine and Health Sciences, Wollo University will review and approved this research proposal project. If you want to know more information, you can contact the following individuals and you may ask at any time as you want: -

Principal investigator: Alebachew Kassa

Email Address; alebachewkassa66@gmail.com; Mobile: +251919996612

Advisors: 1.Mr. Fasil Walelign (Assistant professor)

Email address; fasilowa2@gmail.com, Mobile: +251935014875

2. Mr. Sisay Eshete (Mph)

Email address; sisliyu21@gmail.com, Mobile: +251911002043

Consent form

I, (study participant) have been already understand the purposes, procedures, benefit and risks of this research project as described within it. And also, I understand that all the information regarding to me will be keep confidentially.

1. I freely agree to participate in this research project, as described.
2. I do not agree to participate in this research project

Interviewer name: ________________________ signature___________ Date: __________

Supervisor Name: _______________________signature_____________ Date: __________

1. Completed 2. Partially responded 3.Refused 4. Other, specify

## Annex.II: self-adminstrative questionare

**Part I. Socio-Demographic characteristics**

Dear participant, the following questions are targeted to differentiate your socio-demographic characteristics. Please circle the best proper choice of answer code.

| **No** | **Question** | **Answer options and codes** | **Remark** |
| --- | --- | --- | --- |
|  | Sex | 1. Male 2. Female |  |
|  | Age in years | __________years |  |
|  | Experience in years | -------------Years |  |
|  | Marital status | 1. Single 2. Married 3. Divorced 4. Widowed |  |
|  | Profession | 1. Medical doctor 2. Medical laboratory 3. Nursing 4. midwifery 5. Pharmacy 6. cleaner 7. Other (write name)………… |  |
|  | Educational level | 1. Diploma 2. First degree 3. Second degree and above 4. other |  |
|  | Working department/section | 1.obs/gyn  2. medical ward  3. surgical ward  4. pharmacy  5. laboratory  6. emergency  7. cleaner  8. other |  |

**Part-2. Health institution (health care facility) related characteristics (Give response by "*circling***" the number)

| S/  No | Question related to institution factors | Response | Remark |
| --- | --- | --- | --- |
| 201 | Is there enough supplies to apply standard precautions? (If no skip to 203) | 1.Yes  2. No |  |
| 202 | If "1" ,what are they? | 1.water  2.Soap  3.Alcohol  4.others |  |
| 203 | Did you take training on standard precautions? (If No skip to 205) | 1.Yes  2.No |  |
| 204 | If" 1 "on which standard precautions did you take training? | 1. Hand hygiene  2. Personal protective equipment  3. Safe injection practices  4.Handling and disposing sharps  5.instrument processing and waste managements |  |
| 205 | Is there M&E on standard precautions? | 1. Yes 2. No |  |
| 206 | Do you have infection prevention guidelines avaliable in your department. | 1. Yes 2. No |  |

**Part-3.Individual characteristics (Give response by "*circling*" the number) Part II. Knowledge questions**

Dear participants, the following questions are the target to see your knowledge of safety precautions.Please circle the best choice of your answer code.

| **No** | **Questions** | **Answer options and codes** |
| --- | --- | --- |
| 301 | Have youheard about infection prevention? | 1. Yes 2. No |
| 302 | Do you think that gloves cannot provide complete protection against acquiring infection? | 1. Yes 2. No |
| 303 | Do you think that health care- associated pathogens can be found on normally intact patient skin ? | 1. Yes 2. No |
| 304 | Washing your hands with soap or an alcohol based antiseptic decreases the risk of transmission of hospital acquired pathogens | 1. Yes 2. No |
| 305 | Use of an alcohol based antiseptic for hand hygiene is as effective as soap and water if hands are not visibly dirty | 1. Yes 2. No |
| 306 | Gloves should be worn if blood or body fluid exposure is anticipated | 1. Yes 2. No |
| 307 | There is need to wash hands before doing procedures that do not involve body fluid | 1. Yes 2. No |
| 308 | Can you not wear the same pair of gloves for multiple patients as long as there is no visible contamination on the gloves? | 1. Yes 2. No |
| 309 | Do you know about color coding segregation of healthcare wastes? | 1. Yes 2. No |
| 310 | How maximum full should be the safety box containing sharp medical supplies? | 1. 1/2 full 2. 3/4 full 3. Full 4. I don't know |
| 311 | According to the World Health Organization guideline, what is the maximum delay to start HIV post-exposure prophylaxis? | 1. 24 hours 2. 48 hours 3. 72 hours 4. I don't know |
| 312 | Is there any health hazard associated with healthcare wastes? | 1. Yes 2. No 3. I don't know |
| 313 | Does wearing personal protective equipment reduce the risk of infection? | 1. Yes 2. No 3. I don't know |

**Part III: Attitude questions**

Dear participant, the following questions are targeted to see your attitudes towards safety precautions. Please circle the best choice of your answer code.

| **No** | **Question** | **Answer options and codes** |
| --- | --- | --- |
| 401 | Standard precaution is important for healthcare organizations | 1. Agree 2. Disagree 3. Neutral |
| 402 | Do you believe that standard precaution of infection prevention training is important for healthcare workers | 1. Agree 2. Disagree 3. Neutral |
| 403 | Do you believe PPE protect HCWs from infection | 1. Agree 2. Disagree 3. Neutral |
| 404 | Health care workers are at high risk of infection | 1. Agree 2. Disagree 3. Neutral |
| 405 | All personal protective equipment should be accessible in the working department/ section of the healthcare facility. | 1. Agree 2. Disagree 3. Neutral |
| 406 | Do you agree that in the absence of universal precaution  hospital facilities can be the source of infection | 1. Agree 2. Disagree 3. Neutral |
| 407 | Do you believe needles should not be recapped after use | 1. Agree 2. Disagree 3. Neutral |
| 408 | Sharp materials should be discarded in a safety box | 1. Agree 2. Disagree 3. Neutral |
| 409 | Do you agree that recapping is the cause for needle prick  Injury | 1. Agree 2. Disagree 3. Neutral |
| 410 | If you didn't have taken HBV vaccine before, are you willing to take it? | 1. Agree 2. Disagree 3. Neutral |
| 411 | Wearing facemask and eye goggles during procedures with aerosol production is mandatory | 1. Agree 2. Disagree 3. Neutral |
| 412 | Don't use latex gloves if you have allergy to latex | 1. Agree 2. Disagree 3. Neutral |
| 413 | Telephones and door knobs are not source of infections. | 1. Agree 2. Disagree 3. Neutral |
| 414 | Do keep fingernails trimmed moderately short to reduce the risk of tearing gloves. | 1. Agree 2. Disagree 3. Neutral |

**Part IV Practice questions**

Dear participants, the following questions are the target to see your practices towards safety precautions. Please circle the best choice of your answer code.

| **No.** | **Questions** | **Answer options and codes** | **Remark** |
| --- | --- | --- | --- |
| 501 | I wash hands before touching a patient | 1. Always 2. Sometimes 3. Never |  |
| 502 | I wash hands before clean/aseptic procedure | 1. Always 2. Sometimes 3. Never |  |
| 503 | I wash hands after touching body fluid exposures | 1. Always 2. Sometimes 3. Never |  |
| 504 | I wash hands after touching a patient | 1. Always 2. Sometimes 3. Never |  |
| 505 | I wash hands immediately after removal of gloves | 1. Always 2. Sometimes 3. Never |  |
| 506 | I wash hands between patient contact | 1. Always 2. Sometimes 3. Never |  |
| 507 | I wash hands touching patient surroundings | 1. Always 2. Sometimes 3. Never |  |
| 508 | I protect myself against body fluds of all patients regardless of their diagnosis | 1. Always 2. Sometimes 3. Never |  |
| 509 | I provide care considering all patients as potential infectious. | 1. Always 2. Sometimes 3. Never |  |
| 510 | I wear clean gloves whenever there is possibility of any body fluids | 1. Always 2. Sometimes 3. Never |  |
| 511 | I avoid wearing my gown out of work palace | 1. Always 2. Sometimes 3. Never |  |
| 512 | I wear a waterproof apron whenever there is a possibility of body fluid | 1. Always 2. Sometimes 3. Never |  |
| 513 | I wear eye goggles when indicated | 1. Always 2. Sometimes 3. Never |  |
| 514 | I wear mask when indicated | 1. Always 2. Sometimes 3. Never |  |
| 515 | I wear boots when indicated | 1. Always 2. Sometimes 3. Never |  |
| 516 | I use sterilized all reusable equipment before being used on an other patient | 1. Always 2. Sometimes 3. Never |  |
| 517 | I segregate non-infectious wastes in black colour coded dust bin | 1. Always 2. Sometimes 3. Never |  |
| 518 | I clean and disinfect equipment and environmental surfaces | 1. Always 2. Sometimes 3. Never |  |
| 519 | I segregate infectious medical wastes in yellow colour coded dust bin | 1. Always 2. Sometimes 3. Never |  |
| 520 | I dispose immediately all used needles and syringes in to safety box | 1. Always 2. Sometimes 3. Never |  |
| 521 | I place used sharps in puncture resistant container at point of use | 1. Always 2. Sometimes 3. Never |  |
| 522 | Puncture resistant containers for sharps are disposed of when ¾ full | 1. Always 2. Sometimes 3. Never |  |
| 523 | I recap needles | 1. Always 2. Sometimes 3. Never |  |
| 524 | I bend needles | 1. Always 2. Sometimes 3. Never |  |

## Annex III: Qualitative questionaire: English version

For in-depth interview, the following questions will be asked during the discussion:

**Definition**

1. What does mean standard precaution of infection prevention?

2. what are the components standard precaution of infection prevention?

**benefit**

3. In your own opinion, what do you think are the benefits of using infection prevention standard precaution?

**Motivation**

4.What drives you to comply infection prevention**?**

**Barriers**

5. In your own opinion, what do you think would hinder a health care worker from complying with infection prevention standard precautions?

**Practice**

6. In the past three months do you recall having sustained a needle prick injury? What do you think contributed to the injury?

After sustaining the injury, explain the care you received and how soon was the care implemented?

**Challenges**

7.Think of an incident in the past three months when you did not comply with infection prevention standard precautions? Discribe the situation? Explain the reasons for not complying?

# **Annex IV**: Observational cheklist

Factors influencing compliance with standard precautions of infection prevention amng health care workes working at Dessie comprehensive specialized Hospital.

| Item | Yes | No | Comment |
| --- | --- | --- | --- |
| HCWs practice |  |  |  |
| 1.Hand hygiene |  |  |  |
| Hand hygiene by washing with water and soap or using alcohol based hand rub is done:  Before and after performing any procedures |  |  |  |
| Before putting on gloves and after removing gloves |  |  |  |
| After handling contaminated objects. I.e. used instruments |  |  |  |
| Before preparing medication |  |  |  |
| Wash hands beteewn the patients |  |  |  |
| 2.Use of PPEs |  |  |  |
| The following PPEs, are used as follows: |  |  |  |
| Gloves are worn when contact with blood or body fluids, mucus membranes, non-intact skin or potentially infectious materials anticipated. |  |  |  |
| Gowns are worn during procedure, when contact with blood or body fluid is anticipated and during procedures which are likely to generate splashes or srays of blood or other body fluids. |  |  |  |
| Mouth, nose and eye protection is used when a procedure is likely to generate splashes or sprays of blood or other body fluids. |  |  |  |
| 3.Instrument processing |  |  |  |
| Used instruments are immediately decontaminated after use by soaking in 0.5% chlorine for 10 minutes and washed soapy water, then rinsed in a clean water and dried before sending them for high level disinfection or sterilization |  |  |  |
| 4.Waste management |  |  |  |
| 4.1 Sharps |  |  |  |
| Needles are not recapped or bent after use and both needle and syringes are immediately disposed of in to a puncture resistance container. |  |  |  |
| Puncture resistance containers for sharps are disposed of when ¾ full. |  |  |  |
| 4.2 Solid waste |  |  |  |
| Solid waste is segregated at point of use according to category |  |  |  |
| Contaminated waste is disposed of into a container with a plastic liner. |  |  |  |
| Waste containers are disposed of when ¾ full. |  |  |  |
